# POLICIES FOR SUPPORTING CAREGIVERS OF OLDER ADULTS WITH LONG-TERM CARE NEEDS: A MIXED-METHODS SYSTEMATIC REVIEW

**DOI:** 10.1093/geroni/igae098.3589

**Published:** 2024-12-31

**Authors:** Marco Albertini, Eva Bei, Federico Toth

**Affiliations:** University of Bologna, Bologna, Emilia-Romagna, Italy; University of Bologna, Bologna, Emilia-Romagna, Italy; University of Bologna, Bologna, Emilia-Romagna, Italy

## Abstract

**Background:**

In an era marked by the imperative to restrain costs within health and social care services throughout Europe, informal caregivers of older adults assume a pivotal role as primary providers of long-term care. Nevertheless, there remains a shortage in mapping the various social policies pertaining to both direct and indirect support for informal carers across the various EU member states. Objective: This mixed-method systematic review synthesizes data on care policies related to diverse support forms implemented across EU countries to assist caregivers.

**Methods:**

A comprehensive search strategy was conducted in three electronic databases and grey literature to minimise publication bias.

**Results:**

Preliminary findings of studies published between 2010 and 2023 indicate that various forms and levels of support have been introduced or reformed over the past 13 years across EU countries to support caregivers. Despite the challenges posed by population ageing, there is significant policy development disparity among EU members, with some countries to have established mechanisms aside financial assistance to support carers, while others to only now starting to express interest in developing such services. Financial support, primarily through cash-for-care schemes, emerges as the predominant form of assistance, followed by respite care, while other policies in terms of training and mental health services exhibit a notably lower level of development within the EU social policy landscape.

**Conclusion:**

The review is expected to provide significant implications for policymaking, thoroughly mapping the implementation of diverse social care policies across Europe to ensure the sustainability of informal care.

